# Parasitostatic effect of maslinic acid. I. Growth arrest of *Plasmodium falciparum *intraerythrocytic stages

**DOI:** 10.1186/1475-2875-10-82

**Published:** 2011-04-10

**Authors:** Carlos Moneriz, Patricia Marín-García, Andrés García-Granados, José M Bautista, Amalia Diez, Antonio Puyet

**Affiliations:** 1 Departamento de Bioquímica y Biología Molecular IV, Universidad Complutense de Madrid, Facultad de Veterinaria, E28040 Madrid, Spain; 2 Departamento de Ciencias Morfológicas y Biomedicina, Facultad de Ciencias Biomédicas, Universidad Europea de Madrid, 28640, Madrid, Spain; 3 Departamento de Química Orgánica, Facultad de Ciencias, Universidad de Granada, E18071, Granada, Spain; 4 Instituto de Investigación Hospital 12 de Octubre, Universidad Complutense de Madrid, E28040 Madrid, Spain; 5 Departamento de Bioquímica, Facultad de Medicina, Universidad de Cartagena, Cartagena, Colombia

## Abstract

**Background:**

Natural products have played an important role as leads for the development of new drugs against malaria. Recent studies have shown that maslinic acid (MA), a natural triterpene obtained from olive pomace, which displays multiple biological and antimicrobial activities, also exerts inhibitory effects on the development of some Apicomplexan, including *Eimeria, Toxoplasma *and *Neospora*. To ascertain if MA displays anti-malarial activity, the main objective of this study was to asses the effect of MA on *Plasmodium falciparum*-infected erythrocytes *in vitro*.

**Methods:**

Synchronized *P. falciparum*-infected erythrocyte cultures were incubated under different conditions with MA, and compared to chloroquine and atovaquone treated cultures. The effects on parasite growth were determined by monitoring the parasitaemia and the accumulation of the different infective stages visualized in thin blood smears.

**Results:**

MA inhibits the growth of *P. falciparum *Dd2 and 3D7 strains in infected erythrocytes in, dose-dependent manner, leading to the accumulation of immature forms at IC_50 _concentrations, while higher doses produced non-viable parasite cells. MA-treated infected-erythrocyte cultures were compared to those treated with chloroquine or atovaquone, showing significant differences in the pattern of accumulation of parasitic stages. Transient MA treatment at different parasite stages showed that the compound targeted intra-erythrocytic processes from early-ring to schizont stage. These results indicate that MA has a parasitostatic effect, which does not inactivate permanently *P. falciparum*, as the removal of the compound allowed the infection to continue

**Conclusions:**

MA displays anti-malarial activity at multiple intraerythrocytic stages of the parasite and, depending on the dose and incubation time, behaves as a plasmodial parasitostatic compound. This novel parasitostatic effect appears to be unrelated to previous mechanisms proposed for current anti-malarial drugs, and may be relevant to uncover new prospective plasmodial targets and opens novel possibilities of therapies associated to host immune response.

## Background

Despite the increasing number of synthetic and natural compounds which are reported to inhibit the infective cycle of the malaria-related *Plasmodium *species [1-5] only a few of them have been found to be useful in the treatment and prevention of the disease [6]. Current anti-malarial drugs have an effect on a limited selection of plasmodial targets: inhibition of the conversion of toxic haem to haemozoin in the vacuole (chloroquine, quinine, mefloquine and other alkaloids), the inhibition of synthesis of parasite nucleic acids by hindering of the dihydrofolate pathway (pyrimethamine, sulphadoxine, proguanil), the triggering of oxidative stress (artemisinin and derivatives, primaquine) or inhibition of the mitochondrial electron transport chain (atovaquone). Some drugs may affect multiple processes, like chloroquine, which may also cause oxidative damage at the erythrocytic stage [7]. The widespread use of the most effective and affordable drugs, such as chloroquine and the anti-folate combination pyrimetamine-sulphadoxine, has favoured the appearance of resistant Plasmodium variants making the control of the disease more difficult in endemic areas [8]. Resistance to artemisinin has been also recently reported [9,10]. Alternative existing medications are, on the other hand, virtually unaffordable in the countries most seriously affected, or their efficacy may be compromised in the near future by the emergence of new resistant variants. Hence, the search of new synthetic or natural compounds targeting novel biochemical pathways or cell functions is still an imperative need.

The search of natural products showing anti-malarial activity for its direct use or as leads for new drugs is one of the alternatives which are currently explored [11]. Maslinic acid (2α,3β-dihydroxyolean-12-en-28-oic acid) (MA) is natural olenane-type pentacyclic triterpene found in the olive fruit and can be readily obtained from olive pomace oils [12]. Several pharmacological activities have been reported for this compound, such as anti-tumor [13,14], anti-oxidant [15,16], HIV protease inhibitor [17], antimicrobial [18], vasorelaxation [19], and anti-diabetic action mediated by inhibition of glycogen phosphorylase [20,21] and protein tyrosine phosphatase 1B [22]. In addition, it has been shown that MA displays low toxicity on non-tumoral cells [23,24] indicating that it should be safe for use in humans. Several lines of evidence point to a possible anti-malarial activity of MA. HIV protease inhibitors have been recently shown to hinder the pre-erythrocytic stages of Plasmodium [25]. Anti-parasitic activities of MA were also identified against other Apicomplexans [26]. It has been also proposed that the observed growth inhibition of *Toxoplasma gondii *cultures treated with maslinic acid may be related to inhibition of protease activity, leading to defects in gliding motility and ultrastructural alterations of the parasite [24]. Such a possibility suggests a potential activity of maslinic acid, or structurally related molecules, on parasitic processes different from those targeted by current anti-malarial drugs. In addition, other triterpenoid molecules structurally related to MA display very different cytotoxic and antiparasitic activities. Thus, cucurbitane-type triterpenoids have been recently shown to display anti-malarial activity [27], while ursane-type urosolic acid was reported to inhibit growth of *Trypanosoma cruzi *[28] showing in contrast low anti-plasmodial activity [29]. However, hybrid molecules consisting on ursolic acid bound to pharmacophores showed enhanced inhibitory activity on Plasmodium, presumably by facilitating the access of the bound pharmacophore to haem [30]. Maslinic acid has also been used as core compound to synthesize modified derivatives displaying new activities: binding of heterocyclic rings at MA C-2 and C-3 positions increase significantly the inhibitory activity upon human protein tyrosine phosphatase 1B [22], while attachment of hydrophobic groups at C-28 increase inhibition of human glycogen phosphorylase [31]. Coupling of amino acids or dipeptides at C-28 allowed to retain the anti HIV-1 activity while lowering alongside the cytotoxicity [32]. Should MA show significant anti-malarial activity, it would be feasible to use this molecule as a lead compound for the development of a new family of highly active and low toxic anti-plasmodial drugs.

To explore the suitability of maslinic acid as anti-malarial agent, the activity of this triterpene on cultures of *P. falciparum *3D7 and the chloroquine-resistant Dd2 strains has been tested. As none of the currently used anti-malarial drugs targets plasmodial proteases, MA activity was compared to those of chloroquine and atovaquone as examples of effective drugs which interfere the intra-erythrocytic stage of the parasitic cycle.

## Methods

### Drugs and inhibitors

Maslinic acid (MA) was obtained from pressed fruits of olive (*Olea europaea*) by a method previously published [12]. Chloroquine diphosphate salt was purchased from Sigma-Aldrich. Atovaquone was kindly provided by Glaxo-SmithKline. Stock solutions of atovaquone 30 mM and MA 300 mM were prepared in 100% dimethyl sulfoxide (DMSO). Chloroquine was dissolved in distilled water at 100 mM. All compounds were stored at -20°C until use. For the drug assays, serial dilutions were made in culture medium and added to 96-well culture plates. Control cultures were treated with equivalent amounts of DMSO diluted in culture medium.

### In vitro cultures of *Plasmodium falciparum*

*Plasmodium falciparum *strains Dd2 (clone MRA-150) and 3D7 (clone MRA-102) obtained from the MR4 [33] were used for this study. Erythrocytes were obtained from type A+ human healthy local donor and collected in tubes with citrate-phosphate-dextrose anticoagulant (Vacuette^®^). The culture media consisted of standard RPMI 1640 (Sigma-Aldrich) supplemented with 0.5 % Albumax I (Gibco), 100 μM hypoxanthine (Sigma-Aldrich), 25 mM HEPES (Sigma-Aldrich), 12.5 μg/mL gentamicine (Sigma-Aldrich) and 25 mM NaHCO_3 _(Sigma-Aldrich). Each culture was started by mixing uninfected and infected erythrocytes to achieve a 1 % haematocrit and incubated in 5% CO_2 _at 37°C in tissue culture flasks (Iwaki). The progress of growth in the culture was determined by microscopy in thin blood smears stained with Wright's eosin methylene blue solution (Merck), using the freely available Plasmoscore software [34] to monitor the parasitaemia. The detailed description of the culture and synchronization methods used have been reported previously [35].

### Fluorimetric assays for anti-malarial drug activity

A PicoGreen^® ^microfluorimetric DNA-based assay was used to monitor parasite growth inhibition at different drug concentrations [36]. PicoGreen^® ^(P7589) was purchased to Invitrogen and diluted as indicated by the manufacturer in TE buffer (10 mM Tris-HCl, 1 mM EDTA, pH 7.5). Synchronized rings from stock cultures were used to test 200 μM to 0.1 μM serial dilutions of maslinic acid in 96-well culture microplates. Thus, 150 μL of parasites at 2% haematocrit and 1% parasitaemia were allowed to grow for 48-hour in 5% CO_2 _at 37°C. The parasites were then centrifuged at 600 × g for 10 minutes and re-suspended in saponin (0.15%, w/v in phosphate-buffered saline) to lyse the erythrocytes and release the malaria parasites. To eliminate all traces of haemoglobin the pellet was washed by the addition of 200 μL of PBS followed by centrifugation at 600 × g. The washing step was repeated twice to ensure complete removal of haemoglobin. Finally, pellets were re-suspended in 100 μL of PBS. 100 μL of PicoGreen^® ^diluted in TE were added to each well. Plates were incubated for 30-60 minutes in the dark and the fluorescence intensity was measured at 485 nm excitation and 528 nm emission. Growth inhibition was calculated as previously described [36].

### Assay for haem polymerization

The effect of drugs on haem polymerization was tested *in vitro *as described previously [37]. Briefly, a mixture containing 50 μL of 0.5 mg/ml haem (ferriprotoporphyrin IX chloride from Fluka, HPLC purity > 98%, dissolved in dimethylsulphoxide), 100 μL of 0.5 M sodium acetate buffer pH 4.4 and 50 μL of drug solution or solvent (for control), was incubated in a flat bottom 96-well plate (Costar 3799) at 37°C for 48 h. Microplates were then centrifuged at 3300 × g for 15 min. The supernatant was discarded and the remaining pellet was resuspended with 200 μL DMSO to remove unreacted haemin. The microplate was then centrifuged once again and the supernatant eliminated. The pellet consisting of β-haematin was dissolved in 200 μL of 0.1 M NaOH for spectroscopic quantification at 405 nm. The data were expressed as the percentage of inhibition of haem polymerization calculated by the following equation: % inhibition = 100 × (O.D. control - O.D. drug) / (O.D. control).

### Effect of maslinic acid, chloroquine and atovaquone on *P. falciparum *cultures

Synchronized mature rings (22 h) of *P. falciparum *Dd2 at 1% parasitaemia were incubated for 48 hours to complete an infective cycle in the presence of different concentrations of the drugs, and parasite morphology was evaluated by microscopic analysis of Wright's -stained thin blood smears. Smears from drug-free cultures were used as control.

### Viability of *P. falciparum *cultures after treatment with maslinic acid

Synchronous ring, trophozoite, or schizont-stage *P. falciparum *3D7-infected erythrocytes at 10% parasitaemia were incubated with 100 μM MA for 12 hr. All cultures were started using a single synchronized culture from which aliquots were taken at 12 h (young rings), 24 h (trophozoites) and 36 h (schizonts) and treated with MA for 12 hours, followed by MA removal by multiple washes. Untreated cultures and cultures grown in the presence of MA throughout the whole experiment were run in parallel as controls. Stage-specific development was assessed by examining a minimum of 1,000 parasitized cells on each smear, for differential counting of rings, trophozoites, schizonts, and pyknotic forms whose developmental stage could not be established. The fraction of each group was calculated as a percentage of the total parasitized cells. Parasitaemia was measured by counting 1,000 red cells and is reported as the percent of parasitized erythrocytes. Parasite replication was evaluated from successive parasitaemia measurements at the beginning of each new asexual cycle. Smears were made each 12 h and stained. The cultures were monitored for at least 90 hours, corresponding to approximately two cycles of *P. falciparum*.

## Results

### Activity of maslinic acid on Plasmodium-infected erythrocyte cultures

**Figure 1:**
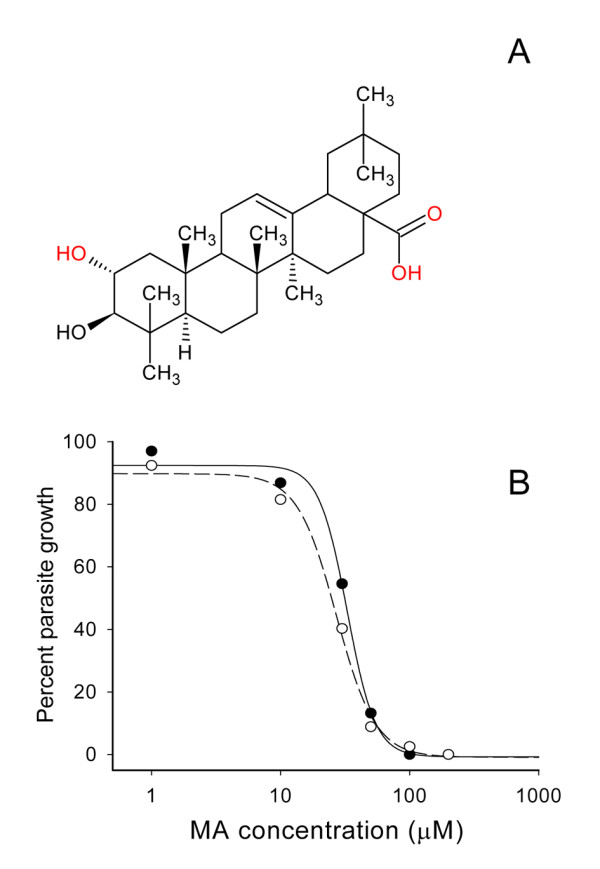
**Dose-response curve of maslinic acid on *P. falciparum *infected erythrocytes**. **A**. Structure of maslinic acid. **B**. *P. falciparum *Dd2 (black circles) and 3D7 (white circles) strains were grown in erythrocyte cultures at 1% initial parasitaemia and incubated with different concentrations of maslinic acid. The DNA content was determined at 48 hours by microfluorimetry. The calculated 50% inhibitory concentration (IC_50_) values were 32 ± 2 for Dd2 and 26 ± 3 μM for 3D7. Results are the mean of three independent cultures.

**Figure 2:**
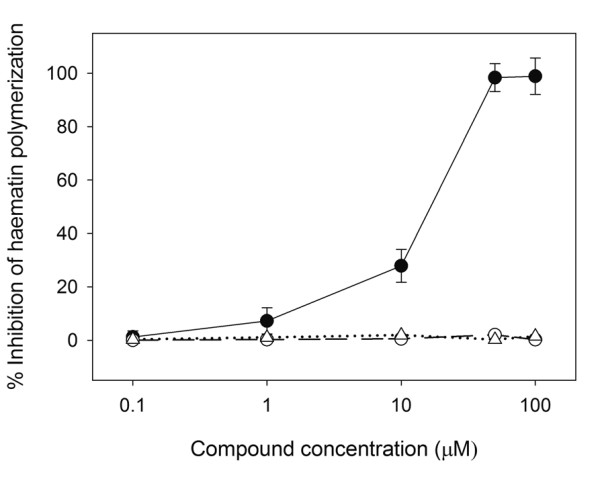
**Assay of β-haematin polymerization**. Haem was incubated in the presence of increasing amounts of maslinic acid (open circle, dashed line), chloroquine (black circle, solid line) and atovaquone (triangle, dotted line) for 48 h, and the formation of β-haematin was determined spectrophotometrically at 405 nm. Results are the mean ± SD of three independent experiments.

The effects of MA on the growth of *P. falciparum *3D7 and the chloroquine resistant strain Dd2 on human erythrocyte cultures were determined by analysing the dose-response curves in the 0.1 to 200 μM range by using microfluorimetric assays on DNA-stained cultures (Figure 1). The cultures showed a dose-dependent inhibitory effect on the parasite growth. The IC50 obtained for Dd2 and 3D7 were, respectively, 32 ± 2 and 26 ± 3 μM. The dose-response curves were almost indistinguishable for both strains. Similar dose-response curves were obtained for chloroquine and atovaquone (not-shown) yielding the following IC50: chloroquine 0.19 μM (Dd2), 0.014 μM (3D7); and atovaquone 0.8 nM (Dd2), 1.1 nM (3D7) The comparable IC50 values for maslinic acid found in chloroquine-resistant and non-resistant strains suggests that this compound may affect plasmodial processes different to those targeted by chloroquine. The main anti-malarial activity of chloroquine appears to involve the inhibition of formation of haemozoin. To discard any interaction of MA with the haemozoin process, a β-haematin polymerization test was performed (Figure 2) which demonstrated that, similarly to atovaquone, MA does not interfere with such parasite activity.

**Figure 3:**
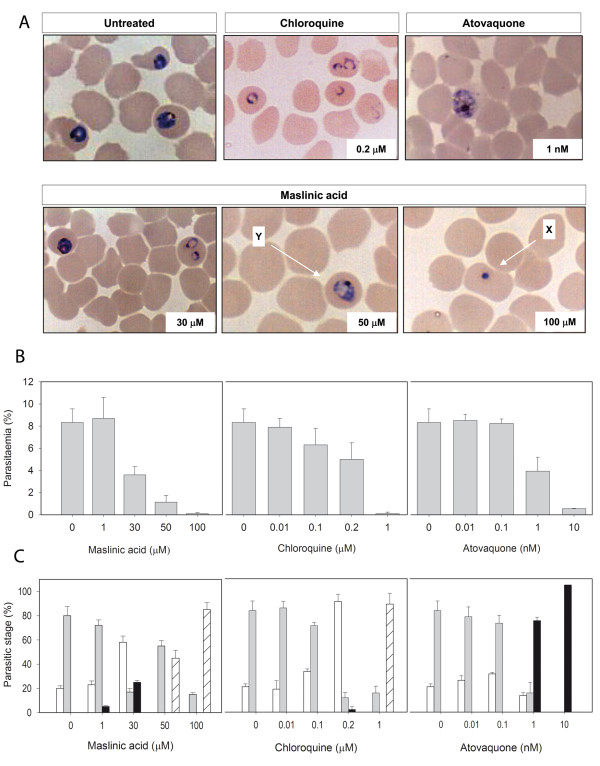
**Dose-dependent effects of maslinic acid on *P. falciparum *cultures compared to chloroquine and atovaquone**. Synchronized cultures of Dd2 at mature ring stage (22 h, 1% parasitaemia) were incubated for 48 hours with the compounds at the indicated concentrations and compared to an untreated control. Cultures were evaluated by microscopic inspection of Wright's-stained thin blood smears. **A**. Microscopic images of *P. falciparum *cultures incubated with different concentrations of maslinic acid, chloroquine and atovaquone. Delayed trophozoites (Y) and pyknotic forms (X) showing condensation of the parasite nucleus are highlighted. **B**. Parasitaemia of the previous cultures after 48 h MA treatment. Results are the mean ± SD of three independent experiments. **C**. Parasitic stages observed in cultures treated with different concentrations of MA, chloroquine and atovaquone for 48 hours. Bars show percentage of rings (white), trophozoites (grey), schizonts (black) and pyknotic (hatched) forms observed in Dd2 cultures treated at the indicated compound concentrations. The percent of each group accounts for the fraction of cells showing every parasite stage from a total of 1000 erythrocytes ± SD. Results are representative of 2 experiments.

To analyse the effects of MA on the maturation of the parasite in the erythrocyte, and compare them to those obtained with chloroquine and atovaquone, synchronized Dd2 infected erythrocytes at mature ring stage (1% parasitaemia) were treated with MA, chloroquine or atovaquone at different concentrations during one infective cycle (48 hours), sampled and examined in thin blood smears. As shown in figure 3, the untreated culture reached 8% parasitaemia and contained predominantly trophozoites at sampling time, indicating that a complete invasive cycle had taken place, producing new trophozoites. Cultures treated with MA at 30 μM (IC50 range) accumulated young rings at the same time, concomitant with a 50% reduction in parasitaemia as compared to the untreated culture. The parasitaemia increase suggests that the parasite was able to invade new erythrocytes, although at lower rates, and the observed accumulation of ring stage may account for both first and delayed second generation parasites. Higher MA concentrations led to the appearance of pyknotic forms and a drastic decrease in parasitaemia (0.1%) suggesting that such dosing was lethal for the parasite. The dose-dependent effect of MA was further demonstrated by a detailed survey of different parasite stages after 48 h treatment (figure 3C). These results suggest a slowdown of *P. falciparum *cell cycle at MA concentrations nearing the IC50. At 30 μM there are still schizonts from the previous cycle, together with new rings which do not progress to trophozoites. Cultures treated with 50 μM MA consisted mainly of trophozoites, likely from the previous cycle as the absence of increase in parasitaemia suggests, as well as pycnotic forms. The lack of ring or schizont stages at this incubation time indicates that at 50 μM MA the parasite was not able to complete the cycle to schizonts and infect new erythrocytes. At their respective IC50, the profiles of parasitic stages observed in the chloroquine and atovaquone cultures were also consistent with a delayed progress of parasite maturation, but showing differences with the profile observed in MA-treated cultures. As shown in the figure 3, 0.2 μM chloroquine-treated cultures accumulated almost exclusively rings, while atovaquone at 1 nM accumulates mainly schizonts, supporting a different mechanism of action for MA as compared to those of chloroquine or atovaquone.

### Effect of MA in the viability of Plasmodium intra-erythrocytic cycle

**Figure 4:**
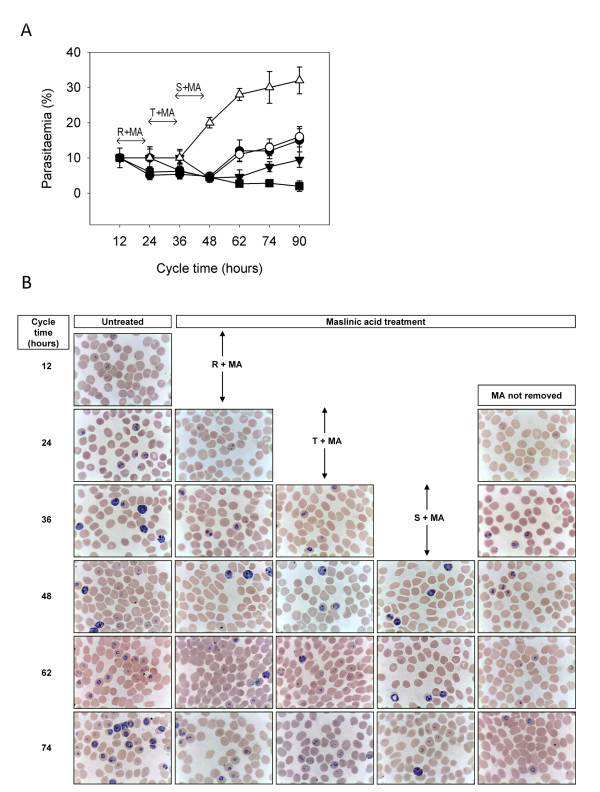
**Effect of transient treatment with MA at different plasmodial stages**. **A**. Development of parasitaemia after maslinic acid treatment at different growth stages of *P. falciparum *3D7. Twelve-hours ring-stage synchronized cultures at 10% parasitaemia were incubated in the presence of MA 100 μM for 12 hours at times corresponding to ring (R+MA), trophozoite (T+MA) and schizont (S+MA) stages. After treatment, MA was removed and parasitaemia was monitored up to 86 h after culture synchronization: untreated (white triangles), R+MA (white circles), T+MA (black circles), S+MA (black triangles), not-removed MA (black squares). Cycle time indicates the elapsed hours of the culture started with synchronized 12 hour-old rings. Results are presented as arithmetic mean of three independent experiments ± SD. **B**. Morphological changes observed in the transiently-treated cultures. Representative light-microscopy fields of cultures subjected to the treatment are shown after Wright's-staining at the times indicated.

**Figure 5:**
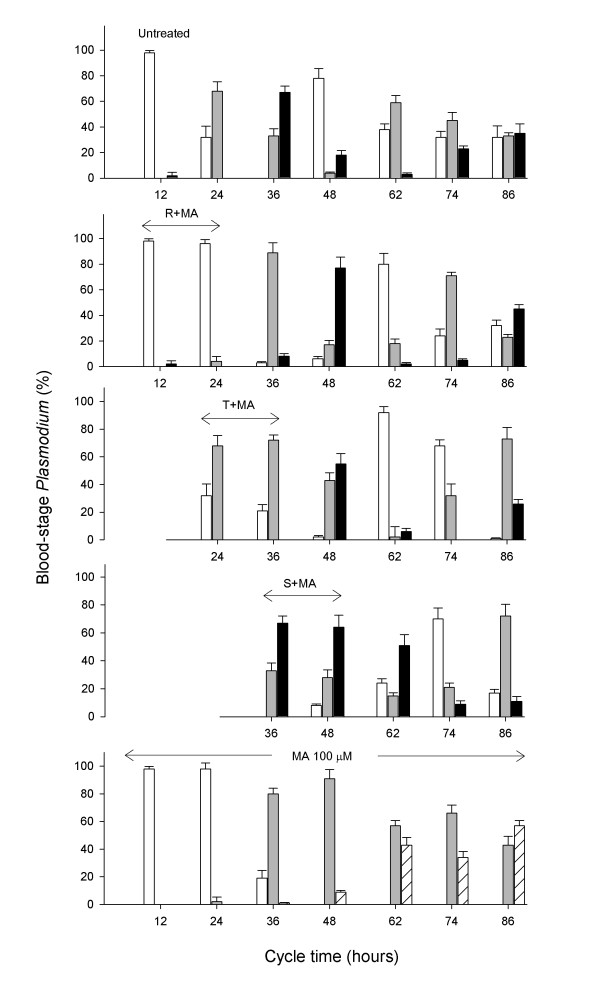
**Effect of maslinic acid treatment at different infection periods in the accumulation of *P. falciparum *erythrocytic stages**. Bars show the percentage of rings (white), trophozoites (grey), schizonts (black) and pyknotic (hatched) forms observed after 12 h incubation with 100 μM MA at ring (R+MA), trophozoite (T+MA) and schizont (S+MA) stages. Data collected by microscopic inspection of Wright's-stained blood smears and compared with controls untreated (top) and cultivated in the presence of 100 μM MA throughout the whole experiment (bottom). The percent of each group accounts for the fraction of cells showing every parasite stage out of a total of 1,000 erythrocytes. Results are representative of two experiments.

As shown above, MA appears to arrest the progress of the *P. falciparum *erythrocytic cycle in a dose-dependent fashion, leading at high doses to the accumulation of trophozoites and pycnotic forms. To ascertain if such effect is reversible and the infection can continue after removal of the compound, MA was added at 100 μM to *P. falciparum *infected erythrocytes at either ring, trophozoite or schizont stages. In this experiment, 100 μM was used as a compromise between 30 μM, at which the effects of MA would not be evident after 12 hours, and 200 μM, at which no viable cells are recovered and, therefore, no recovery of parasite growth would be expected. As similar IC50 for MA were observed for Dd2 and 3D7, the viability assay was performed in 3D7. After 12 h incubation, MA was removed and the parasitaemia was examined every 12 hours thereafter. The results (Figure 4A) showed that all cultures were unable to infect erythrocytes while MA was present, as no increase in parasitaemia at 48 h was observed compared to the untreated culture. All cultures treated transiently with MA resumed the infection thereafter, revealing a parasitostatic effect of MA. Specific treatments at ring and trophozoite stages behave in a similar way, displaying identical recovery rates in parasitaemia values at 62 hours. When maslinic acid was added at the schizont stage, and removed afterwards, the increase in parasitaemia was further delayed, showing a slow recovery at 74 hours. The recovery from the treatment was not immediate at any tested dose. All cultures showed some decrease in parasitaemia after removal of MA until 48 hours, and then a progressive increase of parasitized cells. This behavior can be explained considering the relative high dose of MA used, above the IC50. Under these conditions, a fraction of parasites would be expected to be killed, and only the survivors would be able to resume growth. To examine the effect of these incubations on the intracellular maturation of the parasite, samples were collected at each time and visualized in thin blood smears. As shown in Figure 4B, the 12 hour incubation periods with MA leads to the corresponding delay in the appearance of the subsequent infection stages. This is clearly evidenced in the 48 hours samples, which mostly display newly infected erythrocytes at ring stage in the untreated culture, while treated cultures show schizont-stage parasites arrested in the previous cycle. Figure 5 shows the statistics of the different parasite stages recorded in the same experiment. Ring-stage MA incubated cultures displayed a sharp increase of rings at the end of the treatment (12 h), which subsequently progressed to trophozoites and schizonts, showing an overall 12 hour delay as compared to the untreated control. A similar effect could be recorded both in the trophozoite and schizont-stage treated cultures, with initial accumulation of trophozoites or schizonts, respectively, and the progressive appearance of more mature forms after removal of MA. Remarkably, the recovery of the parasite after incubation with MA appears to be slower when the treatment takes place at mature stages: while rings are not detected 12 h after ring-stage MA treatment, schizonts are still present 24 h after schizont-stage MA treated cultures. These results are consistent with the interference of MA with processes occurring between ring and early schizont stages.

## Discussion

In addition to the broad range of biological activities previously reported for maslinic acid, this compound displays also a significant inhibitory activity on the erythrocytic cycle of *P. falciparum*. Evaluation of IC50 for MA by inhibition of DNA replication in vitro yields a value in the range of 30 μM. This value is even lower than the 54 μM IC50 previously reported for MA on *T. gondii *[24]. The accumulation of rings observed in cultures incubated with this concentration of MA for 48 hours suggest a delay in the progress of the parasite maturation. However, incubation of infected erythrocyte cells with 100 μM MA for 48 hours led to the appearance of non-viable cells. Maslinic acid at concentrations approaching the IC50 appears therefore to slowdown the erythrocytic cycle *of P. falciparum*, while higher doses may induce parasite cell death. In contrast, infected cultures treated with IC50 concentrations of chloroquine or atovaquone led to different parasite stage profiles. Chloroquine 0.2 μM-treated cultures consisted almost exclusively of ring-stage parasites, which may correspond to cell-cycle delayed cells, similarly to the dose-dependent delay recently reported for mefloquine-treated 3D7, W2 and FCB strains [38]. Treatment with atovaquone at 1 nM yields predominantly schizonts. The proposed plasmodial target for atovaquone, an ubiquinone analog, is the mitochondrial *bc1 *cytochrome complex which, once inactivated, leads to mitochondrial membrane depolarization and ultimately, inhibition of DNA synthesis [39]. Such mechanism of action may explain the accumulation of mature forms of the parasite.

Further evidence of the difference between MA and chloroquine inhibitory mechanisms was obtained as MA does not inhibit the accumulation of haematin, thus discarding a possible inhibition of the synthesis haemozoin. In addition the chloroquine-resistant Dd2 strain did not show relevant differences in survival to MA. The resistance to chloroquine has been associated to the acquisition of a mutant transporter PfCRT that is capable of reducing intracellular exposure to the drug [40]. The minor differences observed in the response of Dd2 and 3D7 to MA suggests that PfCRT polymorphisms yielding resistance to chloroquine do not affect the activity of maslinic acid.

The incubation of *P. falciparum *synchronized cultures with MA at different stages of the intra-erythrocytic cycle revealed that the presence of 100 μM MA completely prevents the increase in parasitaemia, leading to parasite cell death if the compound is present up to 86 hours. Remarkably, even at such high concentration, the removal of MA after 12 hour incubation led to the recovery of parasite growth independently of the parasitic stage at treatment, indicating that the effect of this compound on the cell cycle is reversible, although cultures treated with MA at schizont stage showed the slowest rate of recovery to normal parasitaemia. This result is compatible with the possible inhibition of schizont-specific processes. However, the distribution of parasitic stages observed after the removal of MA at different times suggests a more complex picture. Exposure to MA at ring-stage generates accumulation of ring forms and the delay of appearance of trophozoites suggesting that MA inhibition takes place as soon as ring stage. A comparable accumulation of trophozoite and schizont forms are observed after treatments at trophozoite and schizont stages, respectively. Two possible situations may explain this behavior of MA on the infection: either the putative target for MA is a process which takes place along the whole erythrocytic cycle or, alternatively, the compound may display a multiple-target activity, affecting sequentially processes occurring at ring-trophozoite and schizont stages. While ring-trophozoite stages are characterized by massive hemoglobin ingestion, intake of nutrients from the surrounding medium and formation of organelles, schizonts are characterized by DNA replication and the beginning of maturation of merozoite cells with the appearance of merozoite organelles, such as rhoptry and dense granules. Genes expressed preferentially at ring-trophozoite or schizont stages have been identified [41] and may constitute potential targets for the observed activity of MA. Hence, it can be hypothesized that the observed hindering of plasmodial infection progress may be explained as a consequence of proteases and/or phosphatases inhibition, in accordance with previous activities reported for MA and other related triterpenoid molecules [17,22,24,42]. Other possible targets, such as plasmodial topoisomerases [43] or the inhibition of isoprenoid biosynthesis, reported for various terpenes [44], can not be discarded as they may be essential in the processes leading to the development of merozoites.

Maslinic acid displays dose-dependent effect on Plasmodium. Long incubations (48 h) with MA at IC50 range, or short incubations (12 h) with 3X IC50 concentrations lead to the growth arrest of the parasite, while long incubations, even at 1.7X IC50, yield non-viable cells. Unlike antibiotics inhibiting protein synthesis or DNA gyrase activity, which do not lead to phenotypic effects at the end of the first cycle but cause a delayed effect by rendering nonfunctional apicoplasts and schizont arrest in the progeny [45-48], MA appears to exert a non-delayed inhibition of maturation on first-cycle parasites, suggesting different MA-targets to those reported for slow-action antibiotics. The *in vitro *parasitostatic effect of MA was reproduced *in vivo *using a murine malaria model (Moneriz et al, accompanying paper). The use of parasitostatic drugs have not been explored sufficiently as a potential anti-malarial treatment. The interruption of the parasite cycle during the erythrocytic phase should enhance the development of a protective response in the host by extension of the time in which the antigens are exposed to the immune system. Such enhanced acquisition of immunity in mice treated with MA is shown in the accompanying report by Moneriz *et al*. Thus, anti-malarial drugs which specifically interrupt the parasite development maintaining the exposure of antigens may, therefore, become a kind of combined treatment-vaccination approach by using a single compound.

From the results reported in this work, it can be proposed that maslinic acid may be a valuable lead compound to develop a new class of drugs targeting unexploited plasmodial pathways. Following a reverse approach, these molecules would allow to identify and test novel targets for drug development. By comparison of the structures and the activities exhibited by maslinic acid and related molecules, it would be feasible to identify the structural characteristics essential for their anti-parasitic activity and, subsequently, use the natural products as lead compounds for the synthesis of new drugs.

## Conclusions

Maslinic acid emerges as a novel *Plasmodium *parasitostatic natural compound, affecting the whole process of parasite maturation throughout the intraerythrocytic cycle and inhibiting merozoite egression at the IC_50 _range. The growth arrest can be released by elimination of the compound from the culture medium, allowing the parasite to proceed through the subsequent infective stages. Taking into account the parasitostatic effect observed from ring to schizont stages, a multiple-target action on *P. falciparum *is proposed for MA, likely including the inhibition of protease activities essential for growth. The use of parasitostatic agents alone or in combination with other anti-malarial compounds may fuel the development of new therapeutic strategies encompassing the presentation of parasite antigens to the host immune system.

## Conflict of interests

AGG is listed as inventor on a patent owned by the University of Granada related to the use of maslinic acid as antiparasitic agent (date of filing: March 29, 2007; Patent Number: WO/2007/034009). The other authors declare no competing financial interests

## Authors' contributions

CM and PM carried out the laboratory work, contributed in the analysis of data and helped to draft the manuscript. AGG was responsible for maslinic acid production and contributed by sharing knowledge on the compound's biological effects. JMB, AD and AP participated in the analysis and interpretation of the data, and wrote the manuscript. AD and AP conceived and coordinated the study. All authors read and approved the final manuscript.
